# Adherence to Antihypertensive Therapy and Elevated Blood Pressure: Should We Consider the Use of Multiple Medications?

**DOI:** 10.1371/journal.pone.0137451

**Published:** 2015-09-11

**Authors:** Khedidja Hedna, Katja M. Hakkarainen, Hanna Gyllensten, Anna K. Jönsson, Karolina Andersson Sundell, Max Petzold, Staffan Hägg

**Affiliations:** 1 Department of Drug Research/Clinical Pharmacology, Linköping University, Linköping, Sweden; 2 Nordic School of Public Health NHV, Gothenburg, Sweden; 3 EPID Research, Espoo, Finland; 4 Division of Insurance Medicine, Department of Clinical Neuroscience, Karolinska Institutet, Stockholm, Sweden; 5 Department of Clinical Pharmacology and Department of Medical and Health Sciences, Linköping University, Linköping, Sweden; 6 Section of Social Medicine, University of Gothenburg, Gothenburg, Sweden; 7 Centre for Applied Biostatistics, University of Gothenburg, Gothenburg, Sweden; 8 Futurum, Jönköping County Council, Jönköping, Sweden; Hospital de Clínicas de Porto Alegre, BRAZIL

## Abstract

**Background:**

Although a majority of patients with hypertension require a multidrug therapy, this is rarely considered when measuring adherence from refill data. Moreover, investigating the association between refill non-adherence to antihypertensive therapy (AHT) and elevated blood pressure (BP) has been advocated.

**Objective:**

Identify factors associated with non-adherence to AHT, considering the multidrug therapy, and investigate the association between non-adherence to AHT and elevated BP.

**Methods:**

A retrospective cohort study including patients with hypertension, identified from a random sample of 5025 Swedish adults. Two measures of adherence were estimated by the proportion of days covered method (PDC≥80%): (1) Adherence to any antihypertensive medication and, (2) adherence to the full AHT regimen. Multiple logistic regressions were performed to investigate the association between sociodemographic factors (age, sex, education, income), clinical factors (user profile, number of antihypertensive medications, healthcare use, cardiovascular comorbidities) and non-adherence. Moreover, the association between non-adherence (long-term and a month prior to BP measurement) and elevated BP was investigated.

**Results:**

Non-adherence to any antihypertensive medication was higher among persons < 65 years (Odds Ratio, OR 2.75 [95% CI, 1.18–6.43]) and with the lowest income (OR 2.05 [95% CI, 1.01–4.16]). Non-adherence to the full AHT regimen was higher among new users (OR 2.04 [95% CI, 1.32–3.15]), persons using specialized healthcare (OR 1.63, [95% CI, 1.14–2.32]), and having multiple antihypertensive medications (OR 1.85 [95% CI, 1.25–2.75] and OR 5.22 [95% CI, 3.48–7.83], for 2 and ≥3 antihypertensive medications, respectively). Non-adherence to any antihypertensive medication a month prior to healthcare visit was associated with elevated BP.

**Conclusion:**

Sociodemographic factors were associated with non-adherence to any antihypertensive medication while clinical factors with non-adherence to the full AHT regimen. These differing findings support considering the use of multiple antihypertensive medications when measuring refill adherence. Monitoring patients' refill adherence prior to healthcare visit may facilitate interpreting elevated BP.

## Introduction

Hypertension is a major risk factor for cardiovascular morbidity and mortality, affecting more than a quarter of the adult population worldwide [1]. Previous studies have shown that only one third to half of hypertensive patients have adequately controlled blood pressure (BP) [2]. Non-adherence to antihypertensive therapy (AHT) is a significant barrier to effective hypertension management [3]. It is thus a concern given the evidence from clinical trials of the effectiveness of pharmacologic treatments for improving health outcomes and reducing cardiovascular complications [4].

Different methods are used to measure adherence in clinical practice [5]. Registers of filled prescription medicines (refill adherence) is a reliable method to measure adherence in real-life settings and in large populations [6]. This method has been widely used when studying adherence to individual medications or medication classes [7]. However, many patients with hypertension require a multidrug AHT [8]. Therefore, there is a call to consider the use of multiple medications in the therapy regimen when adherence is measured in clinical practice.

Several studies have identified factors associated with non-adherence, such as, younger age [9], presence of comorbidities [10], lower income [11], the multiple medication therapy [12], and being a new user [13]. However, none of previous studies on AHT medications [9,10,12], have differentiated between factors associated with non-adherence to any AHT medication in the therapy and non-adherence to the full AHT therapy regimen, even though they may differ for the distinct types of non-adherence. Therefore, a better understanding of the barriers to adherence to a multidrug AHT therapy in clinical practice is crucial for tailoring interventions to improve adherence [14]. Moreover, few studies have investigated the association between refill non-adherence and elevated BP in real-life settings [7,12]. The objective of this study was therefore to identify factors associated with non-adherence to AHT, considering the use of multiple medications. A secondary objective was to analyze the association between refill non-adherence and elevated BP.

## Methods

### Study population

We identified the study population from a random sample of 5025 adults in the county council of Östergötland, drawn from the Total Population Register of Statistics Sweden. We identified individuals who had in 2007, filled any antihypertensive medication, (defined by Anatomical Therapeutic Chemical system (ATC-codes (S1 Table)) with a diagnosis of essential hypertension from their medical records (International Classification Code (ICD)-10 code: I10), or indication of hypertension from refill data. We excluded persons who had: (i) multi-dose dispensed drugs, as they generally receive their prescribed medications automatically from the pharmacy every two weeks, which creates an artificially regular refill pattern. Prescribed daily doses are also missing for individuals with multi-dose dispensing, which hinders the calculation of refill adherence, (ii) antihypertensive medications purchased for other indication than hypertension, or (iii) non-interpretable prescribed daily doses.

Based on previous studies reporting adherence rates to antihypertensive medications between 50% to 90% [7,12,14], a minimum sample size of 384 was required for a maximum width of +/-5% for the 95% confidence interval.

### Data sources

The Swedish Prescribed Drug Register (SPDR) was used to identify individuals who filled antihypertensive medications. The register has a full coverage of dispensed medications for outpatient use [15]. The Care Data Warehouse of Östergötland was used to identify individuals with diagnosis of essential hypertension. This database includes administrative data on all inpatient and outpatient care provided in the county and is considered to have full coverage. Sociodemographic data were collected from Statistics Sweden and death dates from the Cause of Death Register. Data from the registers were linked using the unique personal identity number [16]. The medical records of the study participants with available BP measurements during healthcare visits in a period of three months in 2008 were reviewed and the BP values extracted.

### Non-adherence to antihypertensive therapy

**Fig 1:**
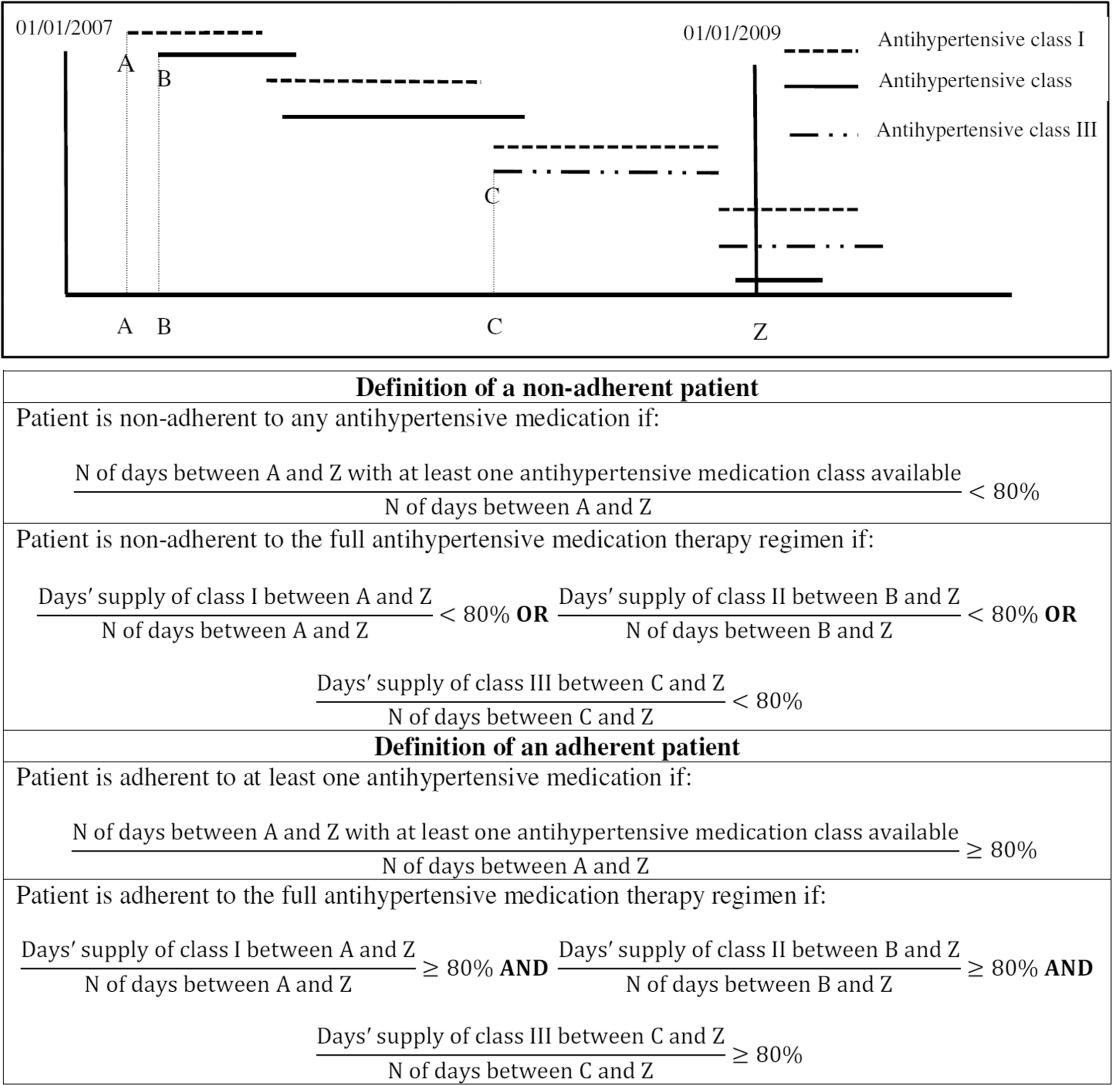
Illustration of measures of adherence to a multiple medication antihypertensive therapy (adapted from Choudhry *et al* ^18^) N: Number. A, B, C: Index dates of antihypertensive classes I, II and III, respectively.

The refill adherence to dispensed antihypertensive medications was measured with the Proportion of Days Covered (PDC) method [17]. We used two definitions of refill adherence to multiple medications: (i) Adherence to any (at least one) antihypertensive medication and, (ii) Adherence to the full AHT regimen [18], which are described below and illustrated in Fig 1. To further clarify the definitions, non-adherence to any (at least one) antihypertensive medication and non-adherence to the full AHT regimen are also illustrated in Fig 1.

To measure the adherence to any antihypertensive medication, we aligned fills of any antihypertensive medication based on dispensing dates and we calculated days’ supply. We calculated the proportion of days a person had at least one antihypertensive medication class available from the date of the first refill in 2007 (index date) until 01/01/2009 or until death. If a prescription days’ supply extended beyond the end of the measurement period, only days for which the medication was available during the measurement period were counted. Patients were considered non-adherent to any antihypertensive medication class if the PDC by any antihypertensive medication during the study period was <80% (Fig 1) [18]. For example, for a patient being treated with three antihypertensive medications, the numerator of the adherence measure was the number of days during which the patient had at least one medication available (either I, II or III).

To measure the adherence to the full AHT regimen, we used the method developed by Choudhry *et al* [18]. We aligned fills of medications within the same class. The index date for each class was the date of the first fill of this medication within the measurement period. If a person filled the same medication prior to the end of the days’ supply of the previous fill, the new medication was assumed to begin after the end of the previous fill. However, if a person switched to another medication from the same class or a fixed combination therapy with a medication from the same class (e.g. switch from Enalapril to a fixed combination of Enalapril and diuretics), the overlapping days were not counted. Patients were considered as non-adherent to the full AHT regimen if at least one antihypertensive medication class in the therapy regimen had a PDC<80% (Fig 1).

### Detection of elevated blood pressure

The medical records were examined by pharmacists who were unaware of the participants’ adherence. Elevated BP was defined as a BP≥140/90mmHg or ≥130/80mmHg in individuals with diabetes mellitus [8]. As we aimed to study the association between non-adherence and elevated BP, we considered the visit with the highest BP value when multiple measurements were available. To assess the sensitivity of measured adherence in predicting BP outcomes, we also assessed the non-adherence 30 days prior to the BP measurement.

### Covariates

Participants’ sociodemographic characteristics included age (<65, 65–79, ≥80 years), sex, highest attained education (mandatory school or less, secondary school, post-secondary or higher) and monthly individual disposable income (in quartiles, the 31^st^ December 2007; Q1 = <Euro 99, Q2 = Euro 100–139, Q3 = Euro 140–201, Q4 = ≥Euro 202. Euro 1 = Swedish Krona 9.3).

Cardiovascular comorbidities were defined with corresponding ICD-10 codes: ischemic heart disease (I20-25), atrial fibrillation (I48), congestive heart failure (I50), previous stroke/transient ischemic attack (I60-69, G45). New users were defined as individuals who did not fill any antihypertensive medication within one year prior to the index date. The number of antihypertensive medications was defined as the maximum number dispensed during the study period. We also described if individuals were dispensed other cardiovascular medications: lipid lowering medications (ATC C10) and anti-diabetic medications (ATC A10). We used the Diagnosis Related Group (DRG) weight as a proxy of severity of comorbidities. Based on the DRG weight, we categorized healthcare use as primary care only, and use of specialized healthcare (inpatient or outpatient) respectively [19].

### Statistical analysis

The characteristics of the study population and those with available BP in the medical records were compared using the two-sample test of proportion for dichotomous variables, Fischer-exact test for categorical variables and Wilcoxon-Mann-Whitney test for continuous, asymmetrically distributed variables.

To study the association between person characteristics and non-adherence to any antihypertensive medication and to the full AHT regimen, we performed multiple logistic regressions, including all covariates mentioned above, except being dispensed other cardiovascular medications because of the high correlation of this variable with existence of cardiovascular comorbidity. Odds ratios (ORs) with 95% confidence intervals (CIs) were calculated for each independent variable in the multiple regression models. We assessed the fit of the model using the Hosmer-Lemeshow test. We also examined the relationship between non-adherence (during the measurement period and 30 days prior to the BP measurement) and elevated BP using multiple logistic regressions with the same variables.

As index date occurred in different dates in 2007, we performed a Poisson regression analysis to assess whether the measurement period of adherence (exposure time) affected our findings. The results from the Poisson regression were in accordance with the logistic regression analyses, and are therefore not presented. Statistical significance was considered for two-sided *p*<0.05. The software SAS version 9.2 (SAS Institute, NC) was used for data management, and Stata version 11.2 (StataCorp, TX) was used for statistical analysis.

### Ethics statement

The study was approved by the Regional Ethical Review Board in Gothenburg (approval reference number: 644–2008). Although personal identity numbers were used for the medical record review, Statistics Sweden replaced the personal identity numbers by a random serial number after the final data linkage and data were analyzed anonymously. According to Swedish legislation, no informed consents from the participants were required, since the retrospective study design did not change the participants’ healthcare and research results were expected to improve the care for future patients [20].

## Results

We identified 1272 individuals who refilled an antihypertensive medication in 2007. We excluded those who had multi-dose dispensed drugs (n = 114), refilled antihypertensive medications for other indications (n = 286), or with non-interpretable dosage instructions (n = 5). The study population included 867 individuals. Of them, 380 individuals had a BP measure in a healthcare visit in a period of three months.

The mean age of the study population was 66.0 years, 52.5% were women, 13.8% new users, and 66.8% had at least two antihypertensive medications (Table 1). Overall, 26.8% of persons had a cardiovascular comorbidity and 70.8% used specialized healthcare. The most common antihypertensive medications were those acting on the renin-angiotensin system (62.0%). Individuals with available BP measures were older, had lower education and income, and used more cardiovascular medications and healthcare resources compared to the whole study population.

**Table 1:** Characteristics of the study population (N = 867) and of individuals with available blood pressure (N = 380).

Persons characteristics	Study population *n* (%)	Individuals with available BP *n* (%)	*p*-value
**Age (years)**			
mean ± SD	66.0±12.1	67.4±11.8	0.003
24–64	388 (44.8)	150 (39.5)	0.018
65–79	354 (40.8)	168 (44.2)	
≥80	125(14.4)	62 (16.3)	
**Sex**			0.426
Female	455 (52.5)	205 (53.9)	
**User profile**			0.492
Prevalent user	747 (86.2)	324 (58.3)	
New user ^a^	120 (13.8)	56 (14.7)	
**Number of dispensed antihypertensive medications** ^b^			<0.0001
1	287 (33.2)	98 (25.8)	
2	302 (34.8)	127 (33.4)	
≥3	278 (32.0)	155 (40.8)	
**Healthcare use** ^c^			
Primary care only	253 (29.2)	78 (20.5)	<0.0001
Specialized care	614 (70.8)	302 (79.5)	
**Cardiovascular comorbidities**	232 (26.8)	137 (36.1)	<0.0001
Congestive heart failure	47 (5.4)	29 (7.6)	
Stroke/transitory ischemic attack	54 (6.2)	31 (8.2)	
Ischemic heart disease	142 (16.4)	85 (22.4)	
Atrial fibrillation	61 (7.0)	42 (11.0)	
**Highest attained education**			0.014
Mandatory school or less	425 (49.8)	201 (52.9)	
Secondary school	306 (35.8)	131 (34.5)	
Post-secondary or higher	123 (14.4)	40 (10.5)	
Missing	13 (1.5)	8 (2.1)	
**Disposable income**			0.012
Quartile 1	195 (22.7)	98 (25.8)	
Quartiles 2–3	483 (56.3)	214 (56.3)	
Quartile 4	180 (21.0)	63 (16.6)	
Missing	9 (1.0)	5 (1.3)	
**Non-adherence**			
Non-adherence to any antihypertensive medications ^d^	110 (12.7)	68 (17.9)	0.718
Non-adherence to the full antihypertensive therapy ^e^	303 (34.9)	152 (40.0)	0.005
**Antihypertensive medications**			
Medications acting on the renin-angiotensin system	539 (62.1)	235 (61.8)	
Beta-blockers	497 (57.3)	223 (58.7)	
Diuretics	414 (47.8)	179 (47.1)	
Calcium channel blockers	322 (37.1)	141 (37.1)	
Anti-adrenergic	7 (0.8)	3 (0.8)	
**Other cardiovascular treatments**			
Lipid lowering	413 (47.3)	206 (54.2)	<0.0001
Anti-diabetic	154 (17.8)	110 (28.9)	<0.0001

BP: Blood pressure; Q1-Q3: First and third quartile; SD: Standard deviation.

^a^ Absence of any antihypertensive medication refill within one year prior to the index date.

^b^ Maximum number of antihypertensive medication classes dispensed during the study period.

^c^ Based on Diagnosis Related Group Weight (DRG weight).

^d^ The proportion of days covered (PDC) with any (at least one) antihypertensive medication available during the study period <80%.

^e^ At least one antihypertensive medication class in the therapy regimen had a PDC <80% during the study period.

In total, 12.7% of the study population was non-adherent to any (at least one) antihypertensive medication and 34.9% non-adherent to the full AHT regimen.

After adjusting for covariates in the multiple models, non-adherence to any antihypertensive medication was higher among persons younger than 65 years (OR 2.75 [95% CI, 1.18–6.43]) and those with the lowest income (OR 2.05 [95% CI, 1.01–4.16]) (Table 2).

**Table 2:** Patient characteristics associated with non-adherence to any antihypertensive medication.

Persons characteristics	Non-adherent*n* (%) ^a^	Crude OR (95% CI)	*p*-value	Adjusted OR ^e^ (95% CI)	*p*-value
**Age (years)**					
<65	68 (17.5)	3.10 (1.45–6.66)	0.004	2.75 (1.18–6.43)	0.020
65–79	34 (9.6)	1.55 (0.70–3.45)	0.279	1.59 (0.67–3.77)	0.291
≥80	8 (6.4)	Reference		Reference	
**Sex**					
Female	54 (11.9)	Reference		Reference	
Male	56 (13.6)	1.17 (0.78–1.74)	0.447	1.33 (0.85–2.09)	0.205
**User profile**					
Prevalent user	84 (11.2)	Reference		Reference	
New user ^b^	26 (21.7)	2.18 (1.34–3.56)	0.002	1.57 (0.92–2.67)	0.093
**Number of dispensed antihypertensive medications** ^c^					
1	60 (20.9)	Reference		Reference	
2	33 (10.9)	0.46 (0.29–0.73)	0.001	0.46 (0.28–0.75)	0.002
≥3	17 (6.1)	0.25 (0.14–0.44)	<0.0001	0.24 (0.13–0.45)	<0.0001
**Healthcare use** ^d^					
Primary care only	30 (11.9)	Reference		Reference	
Specialized care	80 (13.0)	1.11 (0.71–1.74)	0.638	1.27 (0.78–2.07)	0.332
**Cardiovascular comorbidities**					
No	88 (13.9)	Reference		Reference	
Yes	22 (9.5)	0.84 (0.56–1.26)	0.402	1.11 (0.69–1.78)	0.658
**Highest attained education**					
Mandatory school or less	38 (8.9)	Reference		Reference	
Secondary school	45 (14.7)	1.75 (1.11–2.78)	0.016	1.49 (0.91–2.43)	0.109
Post-secondary or higher	22 (12.2)	2.22 (1.26–3.92)	0.006	1.85 (1.00–3.43)	0.050
**Disposable income**					
Quartile 4	23 (12.8)	Reference		Reference	
Quartiles 2–3	60 (12.4)	0.97 (0.58–1.62)	0.902	1.66 (0.94–2.94)	0.081
Quartile 1	25 (12.8)	1.00 (0.55–1.84)	0.990	2.05 (1.01–4.16)	0.045

CI: Confidence interval; OR: Odds ratio.

^a^ The proportion of days with any (at least one) antihypertensive medication available during the study period <80%.

^b^ Absence of any antihypertensive medication refill within one year prior to the index date.

^c^ Maximum number of antihypertensive medication classes concurrently dispensed during the study period.

^d^ Based on Diagnosis Related Group Weight (DRG weight).

^e^ Models were adjusted for number of hypertension medications in the treatment regimen, sex, age, highest attained education, disposable income, DRG weight and the presence of cardiovascular comorbidities. Due to missing data, the multiple logistic regression was performed in 854 individuals.

Non-adherence to the full AHT regimen was higher among new users (OR 2.04 [95% CI, 1.32–3.15]), those who used specialized healthcare (OR 1.63, [95% CI, 1.14–2.32]), and with multiple antihypertensive medications (OR 1.85 [95% CI, 1.25–2.75] and OR 5.22 [95% CI, 3.48–7.83], for 2 and ≥3 antihypertensive medications, respectively) (Table 3).

**Table 3:** Patient characteristics associated with non-adherence to the full antihypertensive therapy.

Persons characteristics	Non-adherent*n* (%) ^a^	Crude OR(95% CI)	*p*-value	Adjusted OR ^e^(95% CI)	*p*-value
**Age (years)**					
<65	142 (36.6)	1.02 (0.67–1.56)	0.904	1.35 (0.83–2.20)	0.211
65–79	116 (32.8)	0.87 (0.56–1.14)	0.511	0.98 (0.61–1.58)	0.946
≥80	45 (36.0)	Reference		Reference	
**Sex**					
Female	163 (35.8)	Reference		Reference	
Male	140 (34.0)	0.92 (0.70–1.22)	0.570	1.02 (0.74–1.40)	0.884
**User profile**					
Prevalent user	250 (33.5)	Reference		Reference	
New user ^b^	53 (44.2)	1.57 (1.06–2.32)	0.023	2.04 (1.32–3.15)	0.001
**Number of dispensed antihypertensive medications** ^c^					
1	60 (20.9)	Reference		Reference	
2	92 (30.5)	1.66 (1.14–2.41)	0.008	1.85 (1.25–2.75)	0.002
≥3	151 (54.3)	4.41 (3.04–6.38)	<0.0001	5.22 (3.48–7.83)	<0.0001
**Healthcare use** ^d^					
Primary care only	68 (26.9)	Reference		Reference	
Specialized care	235 (38.3)	1.69 (1.22–2.33)	<0.001	1.63 (1.14–2.32)	0.007
**Cardiovascular comorbidities**					
Yes	93 (40.1)	1.35 (1.00–1.85)	0.056	0.88 (0.63–1.23)	0.463
No	210 (33.1)	Reference		Reference	
**Highest attained education**					
Mandatory school or less	141(33.2)	Reference		Reference	
Secondary school	107 (35.0)	1.08 (0.79–1.48)	0.614	1.15 (0.82–1.62)	0.421
Post-secondary or higher	47 (38.2)	1.24 (0.82–1.89)	0.301	1.53 (0.96–2.46)	0.076
**Disposable income** ^e^					
Quartile 4	52 (28.9)	Reference		Reference	
Quartiles 2–3	176 (36.4)	1.41 (0.97–2.05)	0.069	1.50 (0.99–2.30)	0.055
Quartile 1	72 (40.0)	1.44 (0.93–2.22)	0.099	1.43 (0.86–2.40)	0.165

CI: Confidence interval; OR: Odds ratio.

^a^ At least one antihypertensive medication class in the therapy regimen had a proportion of days covered <80% during the study period.

^b^ Absence of any antihypertensive medication refill within one year prior to the index date.

^c^ Maximum number of antihypertensive medication classes concurrently dispensed during the study period.

^d^ Based on Diagnosis Related Group Weight (DRG weight).

^e^ Models were adjusted for number of hypertension medications in the treatment regimen, sex, age, highest attained education, disposable income, DRG weight and the presence of cardiovascular comorbidities. Due to missing data, the multiple logistic regression was performed in 854 individuals.

Of the 380 individuals with available BP, 165 (43.4%) had elevated BP. The proportion of persons with elevated BP was higher among those non-adherent to any antihypertensive medication 30 days before the measurement of BP (OR 3.27 [95% CI, 1.27–8.49]) (Table 4). The other measures of non-adherence were not associated with BP control.

**Table 4:** Association between non-adherence and elevated blood pressure (N = 380).

Adherence definition	Persons with elevated blood pressure ^a^ *n* (%)	Crude OR (95% CI)	Adjusted OR ^f^ (95% CI)	*p-*value
**Long term measures of non-adherence**				
Non-adherence to any antihypertensive medication ^b^ (n = 47)	22 (46.8)	1.18 (0.64–2.18)	1.78 (0.90–3.52)	0.098
Non-adherence to the full antihypertensive regimen ^c^ (n = 154)	67 (43.5)	1.02 (0.68–1.55)	1.00 (0.65–1.56)	0.979
**Non-adherence 30 days prior to the blood pressure measure**				
Non-adherence to any antihypertensive medication ^d^ (n = 23)	15 (65.2)	2.61 (1.08–6.33)	3.27 (1.27–8.49)	0.013
Non-adherence to the full antihypertensive regimen ^e^ (n = 109)	50 (45.9)	1.17 (0.75–1.82)	1.04 (0.64–1.70)	0.861

CI: Confidence interval; OR: Odds ratio.

^a^ Defined as a blood pressure (BP)≥140/90mmHg or ≥130/80mmHg in individuals with diabetes mellitus.

^b^ The proportion of days covered (PDC) with any antihypertensive medication available from the index date until the date of BP measure <80%.

^c^ At least one antihypertensive medication class in the therapy regimen had a PDC<80, calculated from the index date until the date of BP measure.

^d^ The PDC with any antihypertensive medication available a month prior to the date of BP measure <80%.

^e^ At least one antihypertensive medication class in the therapy regimen had a PDC<80% a month prior to the date of BP measure.

^f^ Models were adjusted for number of hypertension medications in the treatment regimen, sex, age, highest attained education, disposable income, DRG weight, and the presence of cardiovascular comorbidities.

## Discussion

We found about one-eighth of patients non-adherent to any antihypertensive medication and more than one third non-adherent to the full AHT regimen. Age younger than 65 years and having the lowest income were associated with non-adherence to any antihypertensive medication. While using more healthcare resources, being a new user, and refilling multiple antihypertensive medications were associated with non-adherence to the full AHT regimen. Moreover, non-adherence to any antihypertensive medication a month before a healthcare visit was associated with higher odds of elevated BP. Yet, this association was not significant for long-term measures of non-adherence.

Our study addressed the call to consider the use of multiple antihypertensive medications when measuring refill adherence. To the best of our knowledge, no previous study has distinguished between patient characteristics associated with non-adherence to the full therapy regimen and non-adherence to any medication in the therapy. However, a better understanding of the barriers to adherence in clinical practice is crucial for tailoring interventions to improve adherence.

Our findings add to the previous evidence of younger age being a barrier to adhere to long-term medications [9], that people younger than 65 years are more often non-adherent to any medication. This may be explained by working-age persons’ challenge to include a regular medication intake routine in their daily schedules or by them perceiving treatments for often asymptomatic conditions unnecessary. Emerging technologies have been promoted to improve adherence, especially in those increasingly using new technologies, such as younger adults, but evidence on their long-term efficacy in real-world settings is limited [21].Our findings are also in accordance with previous studies that have found low socioeconomic status associated with non-adherence to medications [11]. This may be explained by multiple factors, including financial barriers. Although the Swedish Pharmaceutical Benefits Scheme has universal coverage independently of persons’ income [22], the commonness of non-adherence to any antihypertensive medication among persons with the lowest income, may indicate a potential for healthcare inequity. In addition, health behavior of people with lower socioeconomic status has been suggested to partly explain non-adherence [23].

Non-adherence to the full AHT regimen among those using specialized healthcare, new users, and those using multiple antihypertensive medications was in accordance with former findings [12,13,24]. Previous studies have reported non-adherence to AHT more common among persons with comorbidities, possibly due to persons with comorbidities prioritizing managing symptomatic conditions over controlling hypertension [10]. Among new users, concerns of dependence and side effects of multiple medications have been reported as the main reasons to discontinue antihypertensive medications [25]. The higher rate of non-adherence to the full therapy regimen among persons with multiple antihypertensive medications may reflect the detrimental effect of multiple medications on adherence and supports decreasing the complexity of AHT to avoid non-adherence and discontinuation.

Our findings are consistent with a large study where participants more likely adhered to at least one antihypertensive medication and less likely to their full therapy [12]. Possibly due to people taking multiple medications having more occasions to refill any medication in their therapy. While some studies have found a better adherence for additional concurrent medications [26], our study highlights the variability in the effect of the number of medication on the overall adherence when applying two different definitions of adherence to a multidrug therapy. More importantly, the extent of disparate interpretations of the effect of multiple medications on the adherence may pose decision-making issues for health practitioners for the management of chronic diseases. Such as, whether a health care provider considers that a patient must adhere to all medications as prescribed, or if he may perceive enough to follow at least one medication in the regimen in order to consider the patient adherent [27].

Nevertheless, in discordance with previous studies [28], we did not find a better adherence among patients with cardiovascular comorbidities. As we did not examine the chronological order of the onset of diseases, non-adherence to antihypertensive medications may have been the cause of other cardiovascular comorbidities in some patients. The level of education was not associated with non-adherence; suggesting that understanding the importance of treatment is probably more important than the level of education [29]. Sex did not either affect adherence and previous findings are contradictory [30,31].

Non-adherence to any antihypertensive medication a month prior to healthcare visit was associated with higher odds of elevated BP. This indicates that monitoring refilled antihypertensive medications prior to healthcare visit may facilitate interpreting observed BP prior to adjusting the therapy regimen. The clinician may attribute elevated blood pressure to therapeutic ineffectiveness and increase the dosage of current medications, or add new medications which may potentially lead to adverse drug reactions. Monitoring any refilled medication, which is a non-invasive method, can for example help the prescriber to distinguish between patients with true resistant hypertension and those with apparent resistant hypertension because of non-adherence to any medication in the therapy [3]. Some studies have also suggested improving the communication between pharmacists, physicians and patients to enhance the rational use and the adherence to long-term medications [32]. However, there is a lack of evidence on methods to introduce refill adherence measurements into clinical practice and their use to improve adherence and health outcomes in chronic care management [33]. The absence of an association between non-adherence to the full therapy regimen a month prior to healthcare visit and elevated BP may be due to the different prescribed medications and that was beyond the scope of this study. Although we did not find an association between long-term non-adherence and elevated BP, this measure represents a healthcare quality indicator to compare across broad population. We should highlight that, similar to previous studies [7,12], less than six out of ten patients adherent to the full therapy reached the target BP, demonstrating a room for improvement in hypertension management and control.

### Strengths and limitations

An important strength of the study was the inclusion of hypertensive patients from a representative sample of the general Swedish population with no selection bias. However, our findings on the association between non-adherence and elevated BP should be interpreted with caution because of the difference in characteristics of those with BP measures and the whole study population. Our findings are not likely to be generalizable to individuals with labile hypertension. However, our method may serve as an impetus for larger studies to better understand the relation between refill adherence and clinical outcomes in real life settings.

Similar to other studies measuring refill adherence, medication consumption was assumed and may overestimate adherence. Nevertheless, previous studies have found that refill adherence is correlated with other measures of adherence [6]. Although our study did not allow assessing primary non-adherence [34], only 3.1% of those with diagnosed essential hypertension did not purchase any medication during the study period. The association of the two measures of refill non-adherence strengthened our study as it allowed combining a sensitive and a specific method to detect non-adherent persons. Non-adherence to any medication may be practically useful for healthcare givers and healthcare systems to screen patients non-adherent to any antihypertensive medications while non-adherence to the full therapy regimen, being more specific, may better detect behavior changes [18]. The method to assess adherence to the full therapy regimen may underestimate adherence when persons switched appropriately to another antihypertensive class. However, there is no gold standard method to assess the adherence to a full multidrug therapy for chronic conditions. This method was considered as appropriate and associated with health outcomes in a previous study encompassing a large study population [35].

While we adjusted our regression models with known factors associated with medication non-adherence, our study design did not allow investigating other factors, such as patients’ understanding of side effects and patients’ relation with healthcare providers [36]. We were also unable to explore the “healthy adherer bias” [37], because healthy behaviors, such as smoking and overweight, were poorly reported in the medical records. Other possible sources of bias include the use of ICD codes to identify comorbidities, and differing practices to measure BP, although BP was assessed according to common practice in primary care. Moreover, the small sample size did not allow adjusting for a larger number of variables as, non-cardiovascular comorbidities. Healthcare resource use was assessed simultaneously with adherence, hindering establishing temporality as non-adherence might have led to increasing morbidity and higher medical care use in some patients.

## Conclusion

In conclusion, sociodemographic factors, i.e. working age and having the lowest income, were associated with non-adherence to any antihypertensive medication while clinical factors were associated with non-adherence to the full AHT regimen. Different factors being associated with distinct types of non-adherence support considering the use of multiple antihypertensive medications when measuring adherence. Distinguishing between patient characteristics associated with non-adherence to any medication in the therapy and non-adherence to the full therapy helps to enhance the understanding of non-adherence to AHT. Moreover, non-adherence to any antihypertensive medication a month before a healthcare visit was associated with elevated BP and suggests that monitoring patients’ refill adherence prior to healthcare visit may facilitate interpreting elevated BP.

## Supporting Information

S1 Table Refilled antihypertensive medications. ATC: Anatomical Therapeutic Chemical classification.
(DOCX)
