# Supplementary material for: Adherence to Antihypertensive Therapy and Elevated Blood Pressure: Should We Consider the Use of Multiple Medications?
Source: PLoS One. 2015 Sep 11;10(9):e0137451. doi: 10.1371/journal.pone.0137451 (PMC4567373; doi:10.1371/journal.pone.0137451)
Supplement: S1 Table — ATC: Anatomical Therapeutic Chemical classification. (DOCX) [file pone.0137451.s001.docx]

**S1 Table. Refilled antihypertensive medications**

| **Class** | **Pharmacological subgroup** | **ATC** | **International Nonproprietary Names** |
| --- | --- | --- | --- |
| Anti-adrenergic |  | C02AC05 | [Moxonidine](http://www.whocc.no/atc_ddd_index/?code=C02AC05&showdescription=yes) |
|  |  | C02CA04 | [Doxazosin](http://www.whocc.no/atc_ddd_index/?code=C02CA04&showdescription=yes) |
| Diuretics | Low ceiling diuretics | C03AA01 | [Bendroflumethiazide](http://www.whocc.no/atc_ddd_index/?code=C03AA01&showdescription=yes) |
|  |  | C03AA03 | [Hydrochlorothiazide](http://www.whocc.no/atc_ddd_index/?code=C03AA03&showdescription=yes) |
|  |  | C03AB01 | [Bendroflumethiazide and potassium](http://www.whocc.no/atc_ddd_index/?code=C03AB01&showdescription=yes) |
|  | High ceiling diuretics | C03CA01 | Furosemide |
|  |  | C03CA04 | [Torasemide](http://www.whocc.no/atc_ddd_index/?code=C03CA04&showdescription=yes) |
|  | [Potassium](http://www.whocc.no/atc_ddd_index/?code=C03D) sparing agents | C03DA01 | Spironolactone |
|  |  | C03DA04 | [Eplerenone](http://www.whocc.no/atc_ddd_index/?code=C03DA04&showdescription=yes) |
|  |  | C03DB01 | Amiloride |
|  | Fixed [combination](http://www.whocc.no/atc_ddd_index/?code=C03EA) of two diuretics | C03EA01 | [Hydrochlorothiazide and potassium-sparing agents](http://www.whocc.no/atc_ddd_index/?code=C03EA01&showdescription=yes) |
| Beta blockers (BB) |  | C07AA03 | [Pindolol](http://www.whocc.no/atc_ddd_index/?code=C07AA03&showdescription=yes) |
|  |  | C07AA05 | [Propranolol](http://www.whocc.no/atc_ddd_index/?code=C07AA05&showdescription=yes) |
|  |  | C07AA07 | [Sotalol](http://www.whocc.no/atc_ddd_index/?code=C07AA07&showdescription=yes) |
|  |  | C07AB02 | [Metoprolol](http://www.whocc.no/atc_ddd_index/?code=C07AB02&showdescription=yes) |
|  |  | C07AB03 | [Atenolol](http://www.whocc.no/atc_ddd_index/?code=C07AB03&showdescription=yes) |
|  |  | C07AB07 | [Bisoprolol](http://www.whocc.no/atc_ddd_index/?code=C07AB07&showdescription=yes) |
|  |  | C07AG02 | [Carvedilol](http://www.whocc.no/atc_ddd_index/?code=C07AG02&showdescription=yes) |
|  | BB and CCB | C07FB02 | [Metoprolol and felodipin](http://www.whocc.no/atc_ddd_index/?code=C07FB07&showdescription=yes) |
| Calcium channels blockers (CCB) |  | C08CA01 | Amlodipine |
|  |  | C08CA02 | Felodipine |
|  |  | C08CA03 | [Isradipine](http://www.whocc.no/atc_ddd_index/?code=C08CA03&showdescription=yes) |
|  |  | C08CA05 | [Nifedipine](http://www.whocc.no/atc_ddd_index/?code=C08CA05&showdescription=yes) |
|  |  | C08CA13 | [Lercanidipine](http://www.whocc.no/atc_ddd_index/?code=C08CA13&showdescription=yes) |
|  |  | C08DA01 | Verapamil |
|  |  | C08DB01 | [Diltiazem](http://www.whocc.no/atc_ddd_index/?code=C08DB01) |
| Medications acting on the Renin-Angiotensin system | Angiotensin converting enzyme inhibitor (ACEI) | C09AA01 | Captopril |
|  |  | C09AA02 | Enalapril |
|  |  | C09AA03 | Lisinopril |
|  |  | C09AA05 | Ramipril |
|  |  | C09AA08 | [Cilazapril](http://www.whocc.no/atc_ddd_index/?code=C09AA08&showdescription=yes) |
|  |  | C09AA09 | [Fosinopril](http://www.whocc.no/atc_ddd_index/?code=C09AA09&showdescription=yes) |
|  | Combination ACEI and diuretics | C09BA02 | [Enalapril and diuretics](http://www.whocc.no/atc_ddd_index/?code=C09BA02&showdescription=yes) |
|  |  | C09BA03 | [Lisinopril and diuretics](http://www.whocc.no/atc_ddd_index/?code=C09BA03&showdescription=yes) |
|  |  | C09BA05 | [Ramipril and diuretics](http://www.whocc.no/atc_ddd_index/?code=C09BA05&showdescription=yes) |
|  |  | C09BA06 | [Quinapril and diuretics](http://www.whocc.no/atc_ddd_index/?code=C09BA06&showdescription=yes) |
|  | [Angiotensin II antagonists](http://www.whocc.no/atc_ddd_index/?code=C09DA) (ARB) | C09CA01 | [Losartan](http://www.whocc.no/atc_ddd_index/?code=C09CA01&showdescription=yes) |
|  |  | C09CA03 | [Valsartan](http://www.whocc.no/atc_ddd_index/?code=C09CA03&showdescription=yes) |
|  |  | C09CA04 | [Irbesartan](http://www.whocc.no/atc_ddd_index/?code=C09CA04&showdescription=yes) |
|  |  | C09CA06 | [Candesartan](http://www.whocc.no/atc_ddd_index/?code=C09CA06&showdescription=yes) |
|  |  | C09CA07 | [Telmisartan](http://www.whocc.no/atc_ddd_index/?code=C09CA07&showdescription=yes) |
|  |  | C09DA01 | [Losartan and diuretics](http://www.whocc.no/atc_ddd_index/?code=C09DA01&showdescription=yes) |
|  | Combination ARB and diuretics | C09DA03 | [Valsartan and diuretics](http://www.whocc.no/atc_ddd_index/?code=C09DA03&showdescription=yes) |
|  |  | C09DA04 | [Irbesartan and diuretics](http://www.whocc.no/atc_ddd_index/?code=C09DA04&showdescription=yes) |
|  |  | C09DA06 | [Candesartan and diuretics](http://www.whocc.no/atc_ddd_index/?code=C09DA06&showdescription=yes) |
|  |  | C09DA07 | [Telmisartan and diuretics](http://www.whocc.no/atc_ddd_index/?code=C09DA07) |

ATC: Anatomical Therapeutic Chemical classification
